# Assessing Tele-Oral Medicine in Saudi Arabia: A Cross-Sectional Study on Specialists’ Experiences and Effectiveness in Oral Healthcare

**DOI:** 10.3390/healthcare11233089

**Published:** 2023-12-02

**Authors:** Sara Akeel, Soulafa Almazrooa, Sarah Alfarabi Ali, Nada A. Alhindi, Sana Alhamed, Osama M. Felemban, Ghada Mansour, Dania Sabbahi, Nada Binmadi, Hani Mawardi

**Affiliations:** 1 Department of Oral Diagnostic Sciences, Faculty of Dentistry, King Abdulaziz University, Jeddah 21589, Saudi Arabia; salmazrooa@kau.edu.sa (S.A.); samali@kau.edu.sa (S.A.A.); nalhendi@kau.edu.sa (N.A.A.); salhamed@kau.edu.sa (S.A.); gmansour@kau.edu.sa (G.M.); nmadi@kau.edu.sa (N.B.); hmawardi@kau.edu.sa (H.M.); 2 Department of Pediatric Dentistry, Faculty of Dentistry, King Abdulaziz University, Jeddah 21589, Saudi Arabia; omfelemban@kau.edu.sa; 3 Department of Dental Public Health, Faculty of Dentistry, King Abdulaziz University, Jeddah 21589, Saudi Arabia; dsabbahi@kau.edu.sa

**Keywords:** oral medicine, oral pathology, oral lesions, teledentistry, tele-oral medicine

## Abstract

Introduction: Teledentistry is an emerging tool to exchange medical information and clinical images to facilitate the diagnosis, prevention, and treatment of oral diseases and patient assurance and education. Considering the shortage of oral medicine specialists in Saudi Arabia, this study aims to assess the experiences of dental specialists with tele-oral medicine and its potential applicability in addressing this shortage. Materials and methods: This was a pilot, cross-sectional study conducted among specialists in the field of oral medicine from January 2020 to March 2020. A total of 16 preselected cases with oral lesions, including clinical history and images, were developed, validated, and shared via email with study participants. Each case included questions on differential diagnosis, provisional diagnosis, and management. The responses were recorded, analyzed, and presented as means and percentages. Results: A total of 49 subjects participated in this study and more than half were under 40 years of age and two-thirds were women. A total of 23 participants had prior experience with tele-oral medicine, mainly via WhatsApp (95.7%), and these cases were received from patients, their families, friends, or other dentists. For all study cases, the correct diagnosis score ranged between 73.50 and 100%, and correct management ranged between 51 and 98%. Conclusions: Tele-oral medicine is an effective tool that may play an important role in patient management in rural regions with a shortage of oral medicine services. Further studies with larger sample sizes and in collaboration with international centers are warranted to confirm these findings.

## 1. Introduction

Teledentistry is an emerging communication tool incorporating digital technology to ensure a wider delivery of dental care to patients in need [1]. Similar to telemedicine, teledentistry permits the exchange of medical information and clinical pictures to facilitate the diagnosis, prevention, and treatment of oral diseases and helps with patient assurance and education [2]. To date, teledentistry has been recognized as an effective tool and is utilized in almost all dental specialties, including oral medicine [1]. In communities with limited access to oral medicine specialists, teledentistry can help patients with oral lesions, specifically in rural areas. This will ensure prioritizing referrals for serious cases that warrant rapid treatment [3]. Additionally, it facilitates the management of simpler local cases without tertiary care facilities [4]. 

Recently, considerable research on teledentistry has been published with a focus on the diagnosis of dental problems and the education of patients through various portals such as emails and phone applications [2,5,6]. However, a limited amount of the literature focuses on tele-oral medicine and its applicability in managing patients with oral lesions [5]. Considering the nature of this specialty, the application of teledentistry would have greater value compared to other dental specialties through avoiding unnecessary clinical visits, managing medically complex patients and/or oral lesions through the prescription of proper medications, or through needed referrals [7]. However, obtaining a detailed patient history should accompany this process, in addition to clinical photos, to mimic the real clinical setting and avoid the risk of false diagnoses or improper management. It is crucial to recognize the limitations of clinical photos. While they can assist in making initial treatment decisions, they cannot offer a definitive diagnosis in some cases. Therefore, specialist involvement is essential for definitive diagnoses in tele-oral medicine. The complexity of the field underscores the importance of expert participation in ensuring reliable provisional diagnoses.

Due to the shortage of oral medicine specialists in Saudi Arabia, this study aims to evaluate the utility and effectiveness of tele-oral medicine, focusing on its role in addressing the challenges of limited access to oral medicine specialists in various settings, including rural areas. It seeks to assess how tele-oral medicine can aid in the management of oral lesions, facilitate prioritized referrals for critical cases, and enhance the delivery of dental care, ultimately contributing to more efficient and accessible oral healthcare [8,9,10]. This paper pioneers the simulation of real clinical oral medicine consultation scenarios, considering selected cases to cover all possible aspects, including patients taking images. This addition will greatly contribute to the literature and facilitate the establishment of this service.

## 2. Materials and Methods

The study was approved by the Ethics committee of King Abdulaziz University Faculty of Dentistry (ID 035-02-18), and informed consent was obtained from all the participants. This was a pilot, cross-sectional study used to evaluate the applicability and reliability of teledentistry in the field of oral medicine in Saudi Arabia. The data were collected from January 2020 to March 2020. The inclusion criteria included certified oral medicine and oral pathology practitioners, periodontists, and/or oral and maxillofacial surgeons (OMFS) who spoke English and were currently practicing in Saudi Arabia. Even with the study focusing on oral medicine cases, other dental specialists were also enrolled in this study, as they are more likely to encounter patients with oral lesions in their practice. Dentists from all other specialties were excluded from this study.

**Figure 1:**
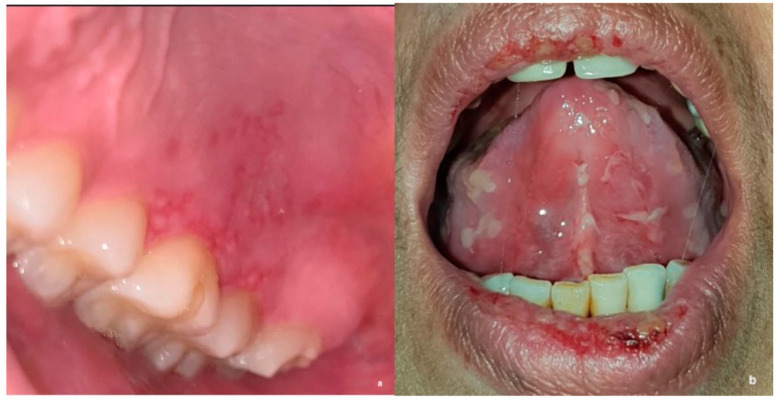
An example of clinical pictures used to represent common disciplines of oral medicine including oral infections (**a**) and vesiculo-bullous lesions (**b**).

**Figure 2:**
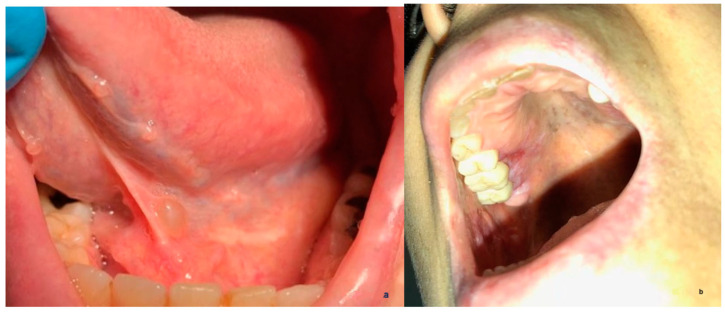
A high-quality picture of a case of a salivary duct cyst taken by an oral medicine specialist (**a**). A low-quality picture of a case of an oral manifestation of systemic lupus erythematosus taken by the patient (**b**).

**Figure 3:**
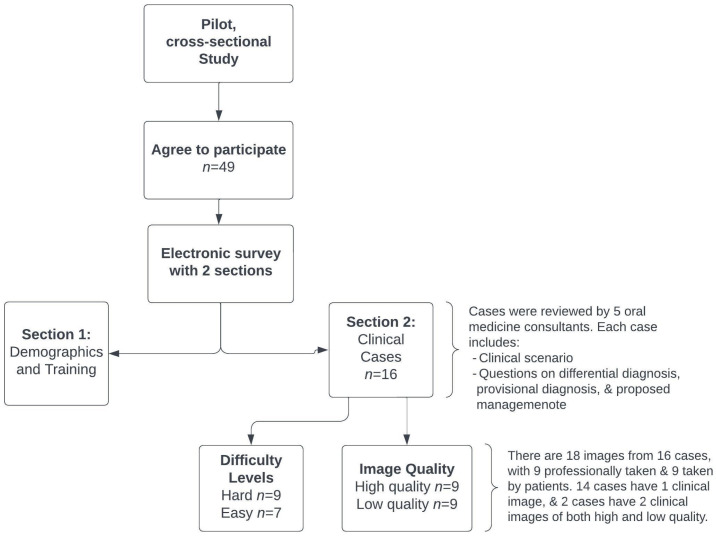
Flow chart illustrating the systematic design of this study.

This study was developed using an electronic survey and included 2 sections. The first section included questions on participants’ demographics, training, and clinical experience. The second section included a series of 16 clinical oral medicine cases, which were carefully selected and reviewed by two oral medicine consultants. Each case was presented with a clinical scenario, followed by questions on differential diagnosis, provisional diagnosis, and proposed management. Study cases were developed to cover the main and common disciplines of oral medicine, including vesiculo-bullous and ulcerative diseases, oral infections, white and red lesions, pigmented and exophytic lesions, and potentially malignant and malignant oral lesions (Figure 1a,b). For management, participants were asked to choose one or more of the following options: (1) patient education and reassurance; (2) medication prescription; (3) a request for further laboratory investigation; and/or (4) a referral to a physician to rule out systemic disease. To assess the role of image quality and case difficulty in generating a correct diagnosis, the study included a total of 18 images for the 16 cases, of which 9 images were taken professionally by dentists while the other 9 lower-quality images were taken by patients (14 cases had 1 clinical image, and 2 cases had 2 clinical images of both high and low quality) (Figure 2a,b). For the purpose of this study, lower image quality was defined as either low resolution, unclear angle, and/or lack of image sharpness. All 16 cases were selected based on binary difficulty levels as either easy (9 cases) or hard (7 cases). For the purpose of this study, the difficulty level was based on either diagnosis commonality and/or clinical presentation (classic or unusual). Determinations were informed by 15–25 years of collective oral medicine experience among senior researchers, considering textbook-defined ‘classic presentations’ and deviations as ‘unusual presentations’ (Figure 3). To assess the importance of providing a detailed patient history, 4 cases (of 16) were initially queried without providing the associated case history, and study subjects were asked for the most likely diagnosis. Afterward, a case history was provided to the study subjects, and the diagnosis was requested again in the following question. To avoid answer modification, the system prevented participants from going back to previous questions. However, no specific time or duration was set for answering all the study questions.

Prior to launching the study, all questions were validated by enrolling a total of 5 oral medicine and oral pathology specialists to provide comments and feedback. Consequently, the study was modified for language, and there was a replacement of clinical images and changes to question style as needed based on the comments and suggestions. Next, the study was distributed using emails and communications platforms (e.g., WhatsApp^®^ version 2.20.19) to all eligible participants identified through official dental societies and organizations in the country. A total of 4 reminders were sent to the participants every 2 weeks to ensure completion of the study.

The total score for each participant was calculated by combining all correct responses. In addition, the final score for each case was calculated by combining the total correct answers for each particular case. A scale ranging from 1 to 5 was used to evaluate participants’ confidence in diagnosing and managing the presented oral lesions. Collected data were analyzed using the Statistical Package for Social Sciences (SPSS) software version 20 (IBM Corp. Released 2011. IBM SPSS Statistics for Windows, Version 20.0. Armonk, NY, USA: IBM Corp.) and reported as frequencies and percentages. An analysis of the image quality and case difficulty effects was completed using the Mann‒Whitney U test.

## 3. Results

A total of 60 subjects met the study eligibility criteria, and 49 responded to all the questions and were included in the analysis (Table 1). Overall, 59.2% of the participants were forty years old or younger, with an age range of 35–40. Females accounted for more than two-thirds of the total subjects (63.3%). In terms of training background, oral medicine specialists accounted for 57.1% of all the participants, followed by oral and maxillofacial pathologists at 20.4% and 22.4% for both oral and maxillofacial surgeons and periodontists. The majority of the participants were affiliated with academic institutes (83.7%), and the rest were based in governmental hospitals and/or private practices.

In total, 26 subjects (53.1%) had no prior experience with tele-oral medicine, 16.3% had managed 1–10 cases, and 30.6% had managed more than 10 cases during their entire dental practice (Table 2). Most managed cases were referred via WhatsApp^®^ version 2.20.19 or higher (95.7%), email (65.2%), and Twitter^®^ version 7.90 or higher (17.4%) by patients themselves, their families, friends (87.0%), or other dentists (82.6%).

As part of the study, three optional questions were asked regarding participants’ perceptions and experiences with tele-oral medicine during the COVID-19 pandemic (Table 3). A total of 28 (57.1%) of the participants responded, and 46.4% indicated that they had utilized tele-oral medicine prior to the COVID-19 pandemic. However, 61.5% of the participants reported a higher demand for tele-oral medicine during and after the pandemic. In addition, 53.5% of the participants had never used tele-oral medicine. However, 13.3% of the group started using tele-oral medicine after the COVID-19 pandemic.

The participants were asked several questions to determine their readiness to implement tele-oral medicine in their daily practice (Table 4). Most participants (79.6%) had completed a rotation in oral medicine or clinical oral pathology as part of their postgraduate training. Furthermore, 85.7% worked in the field of oral medicine or clinical oral pathology. When asked if tele-oral medicine could be utilized in their current clinical practice, 30.6% reported that they already utilized tele-oral medicine, 34.7% were comfortable with starting to use it, 8.2% were against the notion of tele-oral medicine, and 26.5% were undecided.

The data on oral lesions included in the 16 cases of participants’ scores and related to correct diagnosis and confidence in their answers are listed in Table 5. The participants’ mean confidence (scale from one to five) in the overall diagnosis and management of cases ranged from 2.37 ± 1.29 to 4.43 ± 0.98, with correct diagnoses ranging from 73.50% to 100%. For case 4, specifically, 51.0% of the participants correctly identified the diagnosis. In addition, the overall percentage of correct management indicated by participants ranged from 51.0% to 98.0%. In total, 32.7% of the participants specifically advised the correct management for case 16.

An analysis of the participants’ overall correct diagnoses, management, and confidence showed no difference regarding the quality factor of the provided clinical images (Table 6). Compared to easy cases, participants’ scores for proper diagnosis (*p* = 0.012), management (*p* = 0.012), and confidence (*p* = 0.003) were significantly lower in difficult cases, regardless of the quality of the clinical images.

## 4. Discussion

In the last few years, telemedicine has been introduced and utilized in the medical community as an efficient tool to provide health care for patients in need. Historically, dentistry has been a medical field in which treatment must be provided in a clinical setting with the physical presence of patients. Recently, teledentistry has been proposed as an alternative approach for consultations and proper referrals, especially in rural areas and/or when there is limited access to a dental facility [11]. This has been proven to be effective and useful for all dental disciplines, including oral medicine, especially during the COVID-19 pandemic, by reducing the number of referrals since some cases can be managed through virtual consultations [12]. In this study, most participants reported an increase in demand for tele-oral medicine during and after the pandemic. This can be explained by easier access to care, savings in resources, and facilitating more accurate diagnosis and management by specialists via tele-oral medicine.

Oral medicine is a unique discipline in dentistry with a focus on diagnosing a wide range of oral conditions that could be either inflammatory, infectious, autoimmune, or potentially malignant in nature. Residency or clinical rotations in oral medicine provide clinicians with the needed knowledge and skills to accept and manage such cases. In this study, 79.6% of the participants had completed a rotation in oral medicine or clinical oral pathology as part of their postgraduate training and primarily practiced in the field of oral medicine or clinical oral pathology. To reach a correct diagnosis, subjects with one or more oral lesions must be assessed by oral medicine specialists or someone with a background in this field. Considering the universally limited number of specialists in oral medicine, reaching the proper diagnosis of oral lesions can often be challenging [12]. These situations are more likely to take place in rural areas, where patients with oral lesions are commonly seen by general dentists/practitioners with no background in oral medicine. Hence, patients might be referred to tertiary care centers, requiring transportation and additional costs. As a result, many patients end up without treatment due to a lack of awareness regarding follow-up or the presence of access/transportation barriers. For these clinical situations and others, tele-oral medicine carries the benefit of decreasing costs, travel, and waiting time and facilitates access to better care using simple tools such as good lighting and tongue depressors [13,14].

As dental community acceptance of new technologies and advancements in the field may differ, it is important to assess health care providers’ previous experience and readiness to implement tele-oral medicine in their practice. Of the participants, 15 (53.5%) had never used tele-oral medicine before, 34.7% were comfortable implementing it, and 8.2% were against the notion of tele-oral medicine. One way to explain this finding is the concern over false diagnoses without a clinical examination. The other possibility is discomfort with hands-on experience with advanced technological devices and applications.

The literature on tele-oral medicine is sparse. Thus, this study assessed its reliability, effectiveness, and applicability to better understand its role in daily dental practice. Generally, 23 participants (46.9%) had previously managed patients with oral lesions using tele-oral medicine mostly via WhatsApp^®^ version 2.20.19 or higher (95.7%), email (65.2%), and Twitter^®^ version 7.90 or higher (17.4%). As part of the study design, participants were presented with real-life clinical scenarios of oral medicine cases commonly encountered for evaluation, diagnosis, and management. The percentages mentioned in our study (more than 80% correct diagnosis, over 70% correct management) align with recognized cut-off points for high reliability in survey research, as per Taherdoost [15]. These findings support the reliability of tele-oral medicine as an efficient tool. A study was conducted at a Brazilian primary health care center in 2008, in which clinical photos of 25 cases with variable oral lesions and their histories were shared with two oral medicine consultants via email. Similar to this study, the correct diagnosis was reached in 88–95% of cases by consultants [15,16]. In 2013, a similar study was conducted and included clinical photographs of 60 patients with variable oral lesions, including fibroma, lichen planus, leukoplakia, fungal infections, and others. These cases were sent via email to two oral medicine consultants, and a correct diagnosis of 80% was reported by at least one consultant [17]. This was comparable to another study in Italy where the collection of 339 clinical pictures from 96 cases and their clinical information was shared with oral medicine specialists using WhatsApp^®^ version 2.10 or higher, and the specialists were asked for a probable diagnosis when more than 92% were pictures of good quality. The diagnoses of the included cases ranged between traumatic lesions (e.g., fibroma), infectious diseases (e.g., oral candidiasis and herpetic lesions), and immune-mediated diseases (e.g., oral lichen planus, leukoplakia, and squamous cell carcinoma) [18]. The collected responses were compared to a clinicopathological examination and showed an 82% agreement rate. In contrast, a study from Botswana recruited 27 patients with oral lesions for diagnosis and management. At the end of the study, there was a 64% discordance in the management plans of clinicians in remote areas compared to oral medicine specialists. However, the clinicians’ diagnoses were still considered on the specialists’ differential diagnosis list in 91% of cases. In all cases, 82% were referred to specialists when only 30% warranted a referral [3].

In this study, WhatsApp^®^ version 2.20.19 or higher was one method used to share the link to the current study cases and was most utilized by study participants. This app is a free, user-friendly, simple encrypted application that justifies its popularity among users, including health care practitioners, particularly in Saudi Arabia [19]. This was evident in the literature, as out of all communication/social platforms reported in previous studies, WhatsApp^®^ version 2.10 or higher was the most commonly used application for sharing clinical cases [18].

As clinical images used in tele-oral medicine can be received from several sources, the image quality may differ and could have a role in the proper diagnosis of a specific oral finding. Professional images are more likely to occur from medical/dental practitioners compared to someone with minimal or no background in medical photography. A research group from Northern Ireland conducted a study using a triage system for 41 patients with mucosal diseases who were evaluated by general dentists or physicians and obtained high-quality pictures. Later, these images were viewed online by oral medicine specialists for diagnosis, urgency of care, and management, and this also included referrals [20]. At the end of the study, 65% (27/41) of patients had common oral lesions (e.g., frictional keratosis, amalgam tattoo, fibroma, denture granuloma, and candida) that could be virtually managed by an oral medicine specialist and/or referred to the community dentist. At the same time, 20% urgently needed to be seen in the clinic, 80% of these cases required biopsies to rule out malignancies, and 20% had less common oral lesions such as orofacial granulomatosis and sialosis [20]. Another study compared oral lesion diagnosis by an oral medicine specialist during clinical oral examination and then 3 weeks later based on clinical photos taken by patients’ cell phones [21]. A total of 16 patients were included in the study with frictional keratosis, leukoplakia, lichen planus, and submucous fibrosis, in addition to fordyce granules, tori, linea alba, and leukoedema. The sensitivity and specificity of identifying a lesion were reported to be 70% and 100%, respectively. Furthermore, the sensitivity and specificity for categorizing the lesion were reported at 75 and 100%, respectively, and 81% and 100% for the decision of a referral, respectively [21]. In our study, the participants’ correct diagnosis, management, and confidence were not related to the quality of the provided images. However, case difficulty may have a role in the reliability of tele-oral medicine, as scores for proper diagnoses, management, and confidence were significantly lower in difficult cases than in easier cases.

In this study, 91.32% of the participants maintained their initial diagnosis despite additional medical history. The data suggest that the impact of altering initial assessments was relatively modest, with only a minority changing their diagnosis.

In Saudi Arabia, similar to other countries, the COVID-19 pandemic had a major impact on all aspects of life, including access to dental care. Patients with acute oral lesions had to suffer at home during the nationwide mandatory curfew as they had limited access to dental facilities. Even with an easing of the restrictions afterward, many patients were still concerned about visiting their dentists to reduce the risk of contracting the virus, which required the adoption of several modifications to dental visit protocols by dental offices [22]. Additionally, health care practitioners, including dentists, implemented virtual consultations and communication applications to address patients’ chief complaints [23]. Due to a shortage of oral medicine specialists, Saudi Arabia was one country that started using tele-oral medicine even prior to the COVID-19 pandemic to address this demand and patients’ needs [8,9]. However, the demand has increased since, as reported by 16.3% of the study participants, and has reached maximum capacity at each health care institute.

The current study has several limitations. First, the study completion time was longer and ranged between 15 and 20 min in total, which may explain the small number of participants who answered all of the questions. Initially, a total of 20 clinical scenarios were included in this study to cover a wide range of oral conditions, and later this was reduced to a total of 16 to reduce the completion time while also achieving enough power for statistical significance. Second, the field of oral medicine includes a wide range of oral lesions, and only common diagnoses were included in this study. Third, the study included participants with different training backgrounds, which may have accounted for the variation in responses. However, all disciplines of oral medicine, oral pathology, periodontics, and oral and maxillofacial surgery are more likely to encounter and be consulted for oral lesions as well as management compared to other dental specialties.

## 5. Conclusions

Teledentistry is an emerging and unique tool with potential advantages in the growing field of oral medicine. These data demonstrated the acceptable reliability and applicability of tele-oral medicine for the diagnosis of oral lesions and their management, especially in rural areas with limited access to oral medicine services. The expansion and governmental regulations of this tool across the country have a potential benefit that could advance patients’ dental care. Further studies are needed to confirm these findings.

## Figures and Tables

**Table 1:** Demographics and clinical backgrounds of the study participants (*n* = 49 subjects).

Demographics	Categories	*n* (%)
Age	30–40	29 (59.2%)
	41 or more	20 (40.8%)
Gender	Female	31 (63.3%)
	Male	18 (36.7%)
Specialty	Oral medicine	28 (57.1%)
	Oral pathology	10 (20.4%)
	Others *	11 (22.4%)
Years of clinical experience	Less than 5	18 (36.7%)
	5–10	15 (30.6%)
	More than 10	16 (32.7%)
Type of practice	Academic	41 (83.7%)
	Clinic setting	8 (16.3%)

* periodontists and oral and maxillofacial surgeons.

**Table 2:** Participants’ previous experience with tele-oral medicine.

Questions	N	Responses	*n* (%)
Approximately, how many total cases have you managed in your clinical practice using tele-oral medicine?	49	0	26 (53.1%)
		1–10	8 (16.3%)
		More than 10	15 (30.6%)
If yes, what platform did you use? (You can choose more than one answer)	23	WhatsApp	22 (95.7%)
	23	Email	15 (65.2%)
	23	Twitter	4 (17.4%)
	23	Facebook	2 (8.7%)
	23	Instagram	1 (4.3%)
	23	Phone	1 (4.3%)
What was the main source of the received oral medicine consultations? (You can choose more than one answer)	23	Patient or his or her family member or friend	20 (87.0%)
	23	Dentist	19 (82.6%)
	23	Physician	10 (43.5%)

**Table 3:** Participants’ perceptions of tele-oral medicine during the COVID-19 pandemic (*n* = 28 subjects).

Questions (Optional)	Responses	*n* (%)
Have you used tele-oral medicine before the COVID-19 pandemic?	Yes	13 (46.4%)
	No	15 (53.6%)
If yes, has your use of tele-oral medicine increased after the COVID-19 pandemic? (*n* = 13)	Yes	8 (61.5%)
	No	5 (38.5%)
If you answered no to the first question, did you start using tele-oral medicine after the COVID-19 pandemic? (*n* = 15)	Yes	2 (13.3%)
	No	13 (86.7%)

**Table 4:** Readiness of the study participants to implement tele-oral medicine into daily practice (*n* = 49 subjects).

Questions	Responses	*n* (%)
Did you have oral medicine/clinical oral pathology rotation as part of your graduate training program?	Yes	39 (79.6%)
	No	10 (20.4%)
Do you currently practice oral medicine/clinical oral pathology?	Yes	42 (85.7%)
	No	7 (14.3%)
Do you think you can use tele-oral medicine in your current clinical practice?	Already using it	15 (30.6%)
	Yes	17 (34.7%)
	No	4 (8.2%)
	I don’t know	13 (26.5%)

**Table 5:** The overall performance of study participants in diagnosing and managing the provided 16 clinical cases (*n* = 49 subjects).

Case No.	Actual Case Diagnosis	Correct Diagnosis or Differential Diagnosis n (%)	Correct Management n (%)	On a Scale from 1 to 5, How Confident Are You in the Diagnosis and Management of This Case Using Tele-Oral Medicine?
1	Pyogenic granuloma	49 (100%)	46 (93.9%)	3.68 ± 1.16
2	Salivary duct cyst	43 (87.8%)	27 (55.1%)	3.69 ± 1.16
3	Recurrent intraoral herpes	44 (89.8%)	48 (98%)	3.88 ± 1.03
4	Lichen planus	25 (51%)	44 (89.9)	3.27 ± 1.29
5	Pemphigus vulgaris	47 (95.9%)	34 (69.4%)	3.55 ± 1.21
6	Candidiasis	47 (95.9%)	37 (75.5%)	3.96 ± 1.04
7	Chemical burn	48 (98%)	41 (83.7%)	3.84 ± 0.99
8	Recurrent aphthous stomatitis	48 (98%)	47 (95.9%)	4.43 ± 0.98
9	Erythema multiforme	44 (89.8%)	33 (67.3%)	3.37 ± 1.04
10	Leukoplakia	44 (89.8%)	47 (95.9%)	3.90 ± 1.18
11	Squamous cell carcinoma	49 (100%)	45 (91.8%)	4.06 ± 1.30
12	Oral SLE *	42 (85.7%)	25 (51.0%)	3.33 ± 1.27
13	Pyostomatitis vegetans	44 (89.8%)	31 (63.3%)	3.51 ± 1.24
14	Squamous papilloma	46 (93.9%)	43 (87.8%)	3.31 ± 1.25
15	Neutropenic ulcer	39 (79.6%)	36 (73.5%)	3.45 ± 1.08
16	Varices **	36 (73.5%)	16 (32.7%)	3.31 ± 1.29

* systematic lupus erythematosis. ** oral varices is a non-pathologic condition.

**Table 6:** The role of image quality and case difficulty in delivery of correct diagnosis and management of provided cases (*n* = 16 cases).

		Correct Diagnosis or Differential Diagnosis	Correct Management	On a Scale from 1 to 5, How Confident Are You in the Diagnosis and Management of This Case Using Tele-Oral Medicine?
Image quality	Good	89.5 ± 12.9	81.9 ± 14.4	3.7 ± 0.3
	Poor	85.0 ± 11.2	52.9 ± 21.8	3.5 ± 0.4
	*p* value	0.296	0.057	0.521
Case difficulty	Easy	94.8 ± 4.7	86.4 ± 13.7	3.9 ± 0.3
	Difficult	80.8 ± 15.0	63.8 ± 18.2	3.4 ± 0.1
	*p* value	0.012	0.012	0.003

Mann‒Whitney U test.

## Data Availability

The datasets used and/or analyzed during the current study are available from the corresponding author upon reasonable request.
